# Editorial: Brain-computer interface and its applications

**DOI:** 10.3389/fnbot.2023.1140508

**Published:** 2023-02-09

**Authors:** Duo Chen, Ke Liu, Jiayang Guo, Luzheng Bi, Jing Xiang

**Affiliations:** ^1^School of Artificial Intelligence and Information Technology, Nanjing University of Chinese Medicine, Nanjing, China; ^2^School of Computer Science and Technology, Chongqing University of Posts and Telecommunications, Chongqing, China; ^3^National Institute for Data Science in Health and Medicine, Xiamen University, Xiamen, China; ^4^Institute of Mechatronic Systems, Beijing Institute of Technology, Beijing, China; ^5^Magnetoencephalography (MEG) Center, Cincinnati Children's Hospital Medical Center, Cincinnati, OH, United States

**Keywords:** brain-computer interface (BCI), deep learning, machine learning, artificial intelligence, Electroencephalogram (EEG)

Currently, brain-computer interface (BCI) is the research focus and hotspot in the field of neuroscience. Related technologies are widely used in various scenarios such as clinical use, rehabilitation, engineering, and daily life. The BCI uses different brain signals, recording methods, and signal-processing algorithms to build a link between the brain and external software/hardware platforms. With the development of hardware (e.g., BCI chip, wearable device) and algorithms (e.g., machine learning, deep learning), BCI is becoming more practical and stable.

We publish this Research Topic to collect the latest research worldwide in BCI. Researchers from all over the world actively participate and contributed a lot of manuscripts. After carefully and professionally reviewing all submissions, 14 high-quality manuscripts are accepted.

In this topic, several of the contributions focus on the use of deep learning in EEG decoding for BCI, among which convolutional neural network (CNN) is the most widely used. Zhang et al. propose a Multi-Scale 3D-CNN Approach for EEG-based Identity Authentication. The experimental results show that the classification performance of the proposed framework is excellent, and the multi-scale convolution method is effective to extract high-quality identity characteristics across feature domains. Qiu et al. use Electroencephalogram (EEG) and Functional Near Infra-red Spectroscopy (fNIRS) to track the brain activities evoked by neutral and preferred music. The authors conclude that music can promote brain activities, especially in the prefrontal lobe with preferred music. Deng et al. propose SparNet, a CNN composed of five parallel convolutional filters and the Squeeze-and-Excitation Networks (SENet), to learn EEG space-frequency domain characteristics and distinguish between depressive and normal control. The computational results indicate that SparNet achieves a sensitivity of 95.07%, and a specificity of 93.66%. Wang and Cerf combine common spatial pattern (CSP) features and radial basis function neural network (RBFNN) to classify motor imagery EEGs. The algorithm provided high variability within- and across-subjects in EEG-based action decoding. The computational accuracies are higher or close to 90% on two datasets, i.e., BCI competition IV-2a, and -2b. Chen L. et al. transform the EEG signals into symmetric positive definite (SPD) matrices and captures the features of SPD matrices by using CNN. Meta-transfer-learning (MTL) is used to avoid the time-consuming calibration. Chen G. et al. explore the feasibility of an audio-assisted visual BCI speller and a deep learning-based single-trial event-related potentials (ERP) decoding strategy. A spatial-temporal attention-based CNN (STA-CNN) is proposed to recognize the single-trial ERP components. The average classification accuracy of STA-CNN is 77.7% in the EEG dataset recorded from 10 subjects.

Other studies use traditional machine learning and statistical methods for BCI applications in certain modes. Xu et al. use a simulated driving platform with an EEG data collection system for the evaluation of human trust in autonomous vehicles. The graphic theoretical analysis illustrates how human trust varies in EEG under semi-autonomous or fully autonomous driving modes. Wang J. et al. use non-linear dynamics of EEG Signals to Classify primary hand movement intent under opposite hand movement. Their experimental results show significant differences in movement-related cortical potentials between hand movement directions under an opposite hand movement. The results may lay a foundation for the future development of EEG-based human augmentation systems for both the handicapped and the healthy. Massé et al. investigate EEG-based alarm detection in the flight simulator. Cognitive fatigue and cognitive load are manipulated to trigger inattentional deafness, and brain activity is recorded via EEG. The results show that alarm omission and alarm detection can be classified based on time-frequency analysis of EEG. Triana-Guzman et al. combine filter bank CSP (FBCSP) and regularized linear discriminant analysis (RLDA) for decoding EEG rhythms offline and online during motor imagery for standing and sitting. The mean accuracy is higher than 80% in offline analysis, and higher than 90% in online experiments. Song et al. propose a rehabilitative motor imagery BCI system that focuses on rejecting false positive (FP) detection in stroke rehabilitation. A two-phase classifier is used to reject the FP. The algorithm achieved 71.76% selectivity and 13.70% FP rate by using only four EEG channels in the patient group with stroke. Kwon et al. propose a new hybrid visual stimuli for steady-state visual evoked potential (SSVEP)-based BCI, which incorporate various periodic motions into conventional flickering stimuli (FS) or pattern reversal stimuli (PRS). Results demonstrate that FS with sine-wave periodic motion and PRS with square-wave periodic motion could effectively improve the BCI performances compared to conventional FS and PRS.

Finally, two studies provide clinical and human factor applications of magnetoencephalography (MEG) and EEG. Wang Y. et al. classify the MEG data of patients with complex partial seizures (CPS) or simple partial seizures (SPS), using support vector machine. The algorithm obtained a classification accuracy higher than 80%. Cui et al. review the music-emotion recognition and analysis based on EEG signals.

Overall, we hope that this topic can provide some references and novel ideas for researchers in BCI. It should be emphasized that for such a rapidly developing research field, the work that has been done so far is only a drop in the ocean. The manuscripts we collect this time can only be a small leaf in the Amazon rainforest. For BCI, there is still a big gap between the current research and the actual use. Things that need to be solved go far beyond.

## Author contributions

All authors listed have made a substantial, direct, and intellectual contribution to the work and approved it for publication.

